# Supplementary material for: Transcriptome Analysis Reveals Vimentin-Induced Disruption of Cell–Cell Associations Augments Breast Cancer Cell Migration
Source: Cells. 2022 Dec 13;11(24):4035. doi: 10.3390/cells11244035 (PMC9776984; doi:10.3390/cells11244035)
Supplement: Supplementary file 1 [file cells-11-04035-s001.zip › DEGs and enrichment analysis.zip!/DEGs and enrichment analysis/WT_VIMvsVector KEGG/GSEA/WT_VIM/BIOSYNTHESIS_OF_AMINO_ACIDS/BIOSYNTHESIS_OF_AMINO_ACIDS(HSA01230).html]

Details for gene set BIOSYNTHESIS\_OF\_AMINO\_ACIDS(HSA01230)[GSEA]

|  || Dataset | fpkm.sample |
| Phenotype | sample.cls |
| Upregulated in class | WT\_VIM |
| GeneSet | BIOSYNTHESIS\_OF\_AMINO\_ACIDS(HSA01230) |
| Enrichment Score (ES) | 0.6775199 |
| Normalized Enrichment Score (NES) | 1.1745749 |
| Nominal p-value | 0.0 |
| FDR q-value | 0.24099988 |
| FWER p-Value | 0.498 |
Table: GSEA Results Summary

  

Fig 1: Enrichment plot: BIOSYNTHESIS\_OF\_AMINO\_ACIDS(HSA01230)      
 Profile of the Running ES Score & Positions of GeneSet Members on the Rank Ordered List

  

| PROBE | DESCRIPTION (from dataset) | GENE SYMBOL | GENE\_TITLE | RANK IN GENE LIST | RANK METRIC SCORE | RUNNING ES | CORE ENRICHMENT || 1 | ENSG00000233719 | GOT2P3 |  |  | 176 | Infinity | -0.0029 | Yes |
| 2 | ENSG00000178723 | GLULP4 |  |  | 434 | Infinity | -0.0073 | Yes |
| 3 | ENSG00000235371 | AL122018.1 |  |  | 443 | Infinity | -0.0074 | Yes |
| 4 | ENSG00000248415 | GAPDHP61 |  |  | 448 | Infinity | -0.0074 | Yes |
| 5 | ENSG00000228312 | GAPDHP45 |  |  | 1051 | Infinity | -0.0176 | Yes |
| 6 | ENSG00000213352 | GAPDHP44 |  |  | 1394 | Infinity | -0.0233 | Yes |
| 7 | ENSG00000236811 | GAPDHP2 |  |  | 1569 | Infinity | -0.0262 | Yes |
| 8 | ENSG00000218582 | GAPDHP63 |  |  | 1789 | Infinity | -0.0299 | Yes |
| 9 | ENSG00000151224 | MAT1A |  |  | 2414 | 24.571 | 0.0706 | Yes |
| 10 | ENSG00000104524 | PYCR3 |  |  | 2418 | 21.700 | 0.1687 | Yes |
| 11 | ENSG00000250886 | AC010368.1 |  |  | 2590 | 8.334 | 0.2035 | Yes |
| 12 | ENSG00000170950 | PGK2 |  |  | 2705 | 6.804 | 0.2323 | Yes |
| 13 | ENSG00000226468 | AC018641.1 |  |  | 2873 | 5.547 | 0.2546 | Yes |
| 14 | ENSG00000233623 | PGAM1P11 |  |  | 2985 | 4.951 | 0.2751 | Yes |
| 15 | ENSG00000235376 | RPEL1 |  |  | 3218 | 4.194 | 0.2901 | Yes |
| 16 | ENSG00000226784 | PGAM4 |  |  | 3442 | 3.715 | 0.3031 | Yes |
| 17 | ENSG00000236993 | GAPDHP21 |  |  | 3755 | 3.310 | 0.3128 | Yes |
| 18 | ENSG00000228328 | AL158201.1 |  |  | 3810 | 3.266 | 0.3266 | Yes |
| 19 | ENSG00000173599 | PC |  |  | 3844 | 3.214 | 0.3406 | Yes |
| 20 | ENSG00000092621 | PHGDH |  |  | 3864 | 3.194 | 0.3547 | Yes |
| 21 | ENSG00000176974 | SHMT1 |  |  | 4089 | 2.973 | 0.3643 | Yes |
| 22 | ENSG00000273654 | AC020904.2 |  |  | 4160 | 2.890 | 0.3762 | Yes |
| 23 | ENSG00000109107 | ALDOC |  |  | 4255 | 2.815 | 0.3874 | Yes |
| 24 | ENSG00000236056 | GAPDHP14 |  |  | 4529 | 2.637 | 0.3946 | Yes |
| 25 | ENSG00000183010 | PYCR1 |  |  | 4577 | 2.627 | 0.4057 | Yes |
| 26 | ENSG00000111669 | TPI1 |  |  | 4961 | 2.428 | 0.4102 | Yes |
| 27 | ENSG00000285043 | ALDOA |  |  | 4963 | 2.426 | 0.4212 | Yes |
| 28 | ENSG00000161653 | NAGS |  |  | 5021 | 2.392 | 0.4310 | Yes |
| 29 | ENSG00000139410 | SDSL |  |  | 5062 | 2.371 | 0.4410 | Yes |
| 30 | ENSG00000130707 | ASS1 |  |  | 5111 | 2.344 | 0.4508 | Yes |
| 31 | ENSG00000235587 | GAPDHP65 |  |  | 5198 | 2.314 | 0.4598 | Yes |
| 32 | ENSG00000230359 | TPI1P2 |  |  | 5352 | 2.254 | 0.4674 | Yes |
| 33 | ENSG00000152556 | PFKM |  |  | 5463 | 2.212 | 0.4756 | Yes |
| 34 | ENSG00000141959 | PFKL |  |  | 5526 | 2.190 | 0.4844 | Yes |
| 35 | ENSG00000074800 | ENO1 |  |  | 5537 | 2.187 | 0.4941 | Yes |
| 36 | ENSG00000153574 | RPIA |  |  | 5692 | 2.140 | 0.5012 | Yes |
| 37 | ENSG00000021826 | CPS1 |  |  | 5706 | 2.135 | 0.5106 | Yes |
| 38 | ENSG00000237437 | ASS1P12 |  |  | 5842 | 2.081 | 0.5177 | Yes |
| 39 | ENSG00000182054 | IDH2 |  |  | 5915 | 2.058 | 0.5258 | Yes |
| 40 | ENSG00000105552 | BCAT2 |  |  | 5931 | 2.052 | 0.5349 | Yes |
| 41 | ENSG00000163931 | TKT |  |  | 5968 | 2.043 | 0.5435 | Yes |
| 42 | ENSG00000226415 | TPI1P1 |  |  | 6013 | 2.030 | 0.5519 | Yes |
| 43 | ENSG00000182199 | SHMT2 |  |  | 6058 | 2.016 | 0.5603 | Yes |
| 44 | ENSG00000235485 | AL021877.1 |  |  | 6168 | 1.992 | 0.5674 | Yes |
| 45 | ENSG00000101911 | PRPS2 |  |  | 6489 | 1.926 | 0.5707 | Yes |
| 46 | ENSG00000067829 | IDH3G |  |  | 6496 | 1.924 | 0.5793 | Yes |
| 47 | ENSG00000186076 | AC012085.1 |  |  | 6510 | 1.922 | 0.5878 | Yes |
| 48 | ENSG00000101365 | IDH3B |  |  | 6527 | 1.918 | 0.5962 | Yes |
| 49 | ENSG00000067057 | PFKP |  |  | 6865 | 1.848 | 0.5988 | Yes |
| 50 | ENSG00000111640 | GAPDH |  |  | 7102 | 1.792 | 0.6029 | Yes |
| 51 | ENSG00000232630 | PRPS1P2 |  |  | 7244 | 1.764 | 0.6085 | Yes |
| 52 | ENSG00000228232 | GAPDHP1 |  |  | 7284 | 1.757 | 0.6158 | Yes |
| 53 | ENSG00000171314 | PGAM1 |  |  | 7364 | 1.744 | 0.6223 | Yes |
| 54 | ENSG00000120053 | GOT1 |  |  | 8029 | 1.619 | 0.6184 | Yes |
| 55 | ENSG00000100412 | ACO2 |  |  | 8076 | 1.613 | 0.6249 | Yes |
| 56 | ENSG00000062485 | CS |  |  | 8325 | 1.574 | 0.6278 | Yes |
| 57 | ENSG00000067225 | PKM |  |  | 8790 | 1.508 | 0.6267 | Yes |
| 58 | ENSG00000216285 | AC078819.1 |  |  | 8952 | 1.488 | 0.6307 | Yes |
| 59 | ENSG00000274276 | CBSL |  |  | 9052 | 1.473 | 0.6357 | Yes |
| 60 | ENSG00000146733 | PSPH |  |  | 9213 | 1.451 | 0.6396 | Yes |
| 61 | ENSG00000059573 | ALDH18A1 |  |  | 9346 | 1.437 | 0.6438 | Yes |
| 62 | ENSG00000126522 | ASL |  |  | 9354 | 1.436 | 0.6502 | Yes |
| 63 | ENSG00000135821 | GLUL |  |  | 10152 | 1.340 | 0.6427 | Yes |
| 64 | ENSG00000102144 | PGK1 |  |  | 10243 | 1.331 | 0.6472 | Yes |
| 65 | ENSG00000125166 | GOT2 |  |  | 10717 | 1.292 | 0.6450 | Yes |
| 66 | ENSG00000236480 | PKMP1 |  |  | 10940 | 1.272 | 0.6470 | Yes |
| 67 | ENSG00000251013 | GAPDHP62 |  |  | 11001 | 1.265 | 0.6517 | Yes |
| 68 | ENSG00000143811 | PYCR2 |  |  | 11008 | 1.264 | 0.6573 | Yes |
| 69 | ENSG00000255182 | AC084125.2 |  |  | 11073 | 1.258 | 0.6619 | Yes |
| 70 | ENSG00000167701 | GPT |  |  | 11128 | 1.254 | 0.6667 | Yes |
| 71 | ENSG00000171759 | PAH |  |  | 11253 | 1.242 | 0.6702 | Yes |
| 72 | ENSG00000177156 | TALDO1 |  |  | 11258 | 1.241 | 0.6757 | Yes |
| 73 | ENSG00000243989 | ACY1 |  |  | 11504 | 1.227 | 0.6771 | Yes |
| 74 | ENSG00000166123 | GPT2 |  |  | 11800 | 1.201 | 0.6775 | Yes |
| 75 | ENSG00000111674 | ENO2 |  |  | 12605 | 1.131 | 0.6690 | No |
| 76 | ENSG00000108515 | ENO3 |  |  | 13133 | 1.092 | 0.6650 | No |
| 77 | ENSG00000160200 | CBS |  |  | 13246 | 1.083 | 0.6680 | No |
| 78 | ENSG00000218682 | AC064847.1 |  |  | 13289 | 1.079 | 0.6721 | No |
| 79 | ENSG00000168906 | MAT2A |  |  | 14356 | 0.998 | 0.6585 | No |
| 80 | ENSG00000122729 | ACO1 |  |  | 14880 | 0.977 | 0.6541 | No |
| 81 | ENSG00000116984 | MTR |  |  | 15001 | 0.970 | 0.6564 | No |
| 82 | ENSG00000038274 | MAT2B |  |  | 15079 | 0.965 | 0.6595 | No |
| 83 | ENSG00000081181 | ARG2 |  |  | 16308 | 0.899 | 0.6427 | No |
| 84 | ENSG00000234115 | AL138878.2 |  |  | 16413 | 0.893 | 0.6450 | No |
| 85 | ENSG00000197713 | RPE |  |  | 16566 | 0.884 | 0.6464 | No |
| 86 | ENSG00000060982 | BCAT1 |  |  | 17135 | 0.849 | 0.6406 | No |
| 87 | ENSG00000135094 | SDS |  |  | 17241 | 0.842 | 0.6426 | No |
| 88 | ENSG00000116761 | CTH |  |  | 17540 | 0.824 | 0.6413 | No |
| 89 | ENSG00000248180 | GAPDHP60 |  |  | 17753 | 0.813 | 0.6413 | No |
| 90 | ENSG00000220563 | PKMP3 |  |  | 18849 | 0.751 | 0.6261 | No |
| 91 | ENSG00000135069 | PSAT1 |  |  | 18974 | 0.744 | 0.6274 | No |
| 92 | ENSG00000224464 | PGAM1P6 |  |  | 20540 | 0.659 | 0.6038 | No |
| 93 | ENSG00000147224 | PRPS1 |  |  | 21328 | 0.618 | 0.5932 | No |
| 94 | ENSG00000254023 | PKMP4 |  |  | 21382 | 0.615 | 0.5951 | No |
| 95 | ENSG00000219951 | AL391416.1 |  |  | 21405 | 0.615 | 0.5975 | No |
| 96 | ENSG00000257316 | AC087311.1 |  |  | 21602 | 0.602 | 0.5969 | No |
| 97 | ENSG00000188316 | ENO4 |  |  | 22331 | 0.555 | 0.5870 | No |
| 98 | ENSG00000213997 | PGAM1P7 |  |  | 22533 | 0.541 | 0.5861 | No |
| 99 | ENSG00000248498 | ASNSP1 |  |  | 22758 | 0.525 | 0.5847 | No |
| 100 | ENSG00000149925 | ALDOA |  |  | 22828 | 0.519 | 0.5858 | No |
| 101 | ENSG00000070669 | ASNS |  |  | 22892 | 0.515 | 0.5871 | No |
| 102 | ENSG00000255200 | PGAM1P8 |  |  | 23609 | 0.469 | 0.5770 | No |
| 103 | ENSG00000219881 | GAPDHP42 |  |  | 23677 | 0.464 | 0.5780 | No |
| 104 | ENSG00000223922 | ASS1P2 |  |  | 23809 | 0.454 | 0.5778 | No |
| 105 | ENSG00000138413 | IDH1 |  |  | 24628 | 0.388 | 0.5657 | No |
| 106 | ENSG00000244723 | ASLP1 |  |  | 24985 | 0.355 | 0.5613 | No |
| 107 | ENSG00000151005 | TKTL2 |  |  | 25200 | 0.330 | 0.5591 | No |
| 108 | ENSG00000250130 | AC090519.1 |  |  | 25268 | 0.324 | 0.5594 | No |
| 109 | ENSG00000284707 | AC079781.5 |  |  | 25400 | 0.316 | 0.5586 | No |
| 110 | ENSG00000186197 | EDARADD |  |  | 25605 | 0.294 | 0.5565 | No |
| 111 | ENSG00000249840 | GAPDHP76 |  |  | 25966 | 0.249 | 0.5515 | No |
| 112 | ENSG00000118520 | ARG1 |  |  | 26099 | 0.240 | 0.5504 | No |
| 113 | ENSG00000226744 | AC005326.1 |  |  | 26452 | 0.196 | 0.5453 | No |
| 114 | ENSG00000235076 | GAPDHP52 |  |  | 26527 | 0.188 | 0.5449 | No |
| 115 | ENSG00000230274 | PGAM1P3 |  |  | 26853 | 0.139 | 0.5400 | No |
| 116 | ENSG00000215325 | ASS1P10 |  |  | 27366 | 0.000 | 0.5313 | No |
| 117 | ENSG00000216378 | AL080285.1 |  |  | 27525 | 0.000 | 0.5286 | No |
| 118 | ENSG00000213290 | PGK1P2 |  |  | 27802 | 0.000 | 0.5239 | No |
| 119 | ENSG00000229570 | GAPDHP58 |  |  | 28623 | 0.000 | 0.5100 | No |
| 120 | ENSG00000242308 | AC048334.1 |  |  | 28669 | 0.000 | 0.5092 | No |
| 121 | ENSG00000233947 | AL353705.3 |  |  | 28814 | 0.000 | 0.5068 | No |
| 122 | ENSG00000249319 | AC068533.4 |  |  | 29368 | 0.000 | 0.4974 | No |
| 123 | ENSG00000216360 | AL136968.1 |  |  | 29537 | 0.000 | 0.4945 | No |
| 124 | ENSG00000248271 | PGAM1P1 |  |  | 29993 | 0.000 | 0.4868 | No |
| 125 | ENSG00000226443 | GAPDHP32 |  |  | 30070 | 0.000 | 0.4855 | No |
| 126 | ENSG00000239207 | GAPDHP39 |  |  | 30302 | 0.000 | 0.4816 | No |
| 127 | ENSG00000234005 | GAPDHP22 |  |  | 30632 | 0.000 | 0.4760 | No |
| 128 | ENSG00000213449 | GAPDHP28 |  |  | 30637 | 0.000 | 0.4759 | No |
| 129 | ENSG00000235892 | PKMP2 |  |  | 31641 | NaN | 0.4589 | No |
| 130 | ENSG00000243986 | ENO1P3 |  |  | 31897 | NaN | 0.4547 | No |
| 131 | ENSG00000280436 | CR392039.5 |  |  | 31915 | NaN | 0.4544 | No |
| 132 | ENSG00000213158 | GAPDHP36 |  |  | 32057 | NaN | 0.4521 | No |
| 133 | ENSG00000231072 | GAPDHP64 |  |  | 32149 | NaN | 0.4506 | No |
| 134 | ENSG00000257915 | GLULP5 |  |  | 32190 | NaN | 0.4499 | No |
| 135 | ENSG00000036473 | OTC |  |  | 32711 | NaN | 0.4411 | No |
| 136 | ENSG00000226278 | PSPHP1 |  |  | 32840 | NaN | 0.4390 | No |
| 137 | ENSG00000223460 | GAPDHP69 |  |  | 33157 | NaN | 0.4337 | No |
| 138 | ENSG00000234529 | GAPDHP19 |  |  | 33164 | NaN | 0.4336 | No |
| 139 | ENSG00000231900 | GOT2P1 |  |  | 33173 | NaN | 0.4335 | No |
| 140 | ENSG00000215583 | ASS1P6 |  |  | 33476 | NaN | 0.4285 | No |
| 141 | ENSG00000248626 | GAPDHP40 |  |  | 33697 | NaN | 0.4248 | No |
| 142 | ENSG00000233634 | GOT2P5 |  |  | 33771 | NaN | 0.4236 | No |
| 143 | ENSG00000213376 | GAPDHP71 |  |  | 34076 | NaN | 0.4185 | No |
| 144 | ENSG00000248838 | PGAM1P13 |  |  | 34158 | NaN | 0.4171 | No |
| 145 | ENSG00000229551 | GAPDHP17 |  |  | 34280 | NaN | 0.4151 | No |
| 146 | ENSG00000185221 | GAPDHP24 |  |  | 34306 | NaN | 0.4147 | No |
| 147 | ENSG00000234158 | RPEP2 |  |  | 34735 | NaN | 0.4075 | No |
| 148 | ENSG00000226847 | PRPS1P1 |  |  | 34820 | NaN | 0.4061 | No |
| 149 | ENSG00000164708 | PGAM2 |  |  | 35321 | NaN | 0.3977 | No |
| 150 | ENSG00000249489 | GAPDHP70 |  |  | 35408 | NaN | 0.3963 | No |
| 151 | ENSG00000254572 | GLULP3 |  |  | 35625 | NaN | 0.3926 | No |
| 152 | ENSG00000216811 | AL035698.1 |  |  | 35761 | NaN | 0.3904 | No |
| 153 | ENSG00000251031 | AC132803.1 |  |  | 35942 | NaN | 0.3874 | No |
| 154 | ENSG00000249255 | PGAM1P9 |  |  | 36065 | NaN | 0.3854 | No |
| 155 | ENSG00000214563 | GAPDHP15 |  |  | 36081 | NaN | 0.3851 | No |
| 156 | ENSG00000251418 | ASS1P8 |  |  | 36700 | NaN | 0.3747 | No |
| 157 | ENSG00000270978 | AC091982.2 |  |  | 36802 | NaN | 0.3730 | No |
| 158 | ENSG00000227948 | AC003989.1 |  |  | 36998 | NaN | 0.3698 | No |
| 159 | ENSG00000267333 | ASNSP6 |  |  | 37269 | NaN | 0.3652 | No |
| 160 | ENSG00000255353 | AP003057.1 |  |  | 37426 | NaN | 0.3626 | No |
| 161 | ENSG00000216624 | GAPDHP72 |  |  | 37621 | NaN | 0.3594 | No |
| 162 | ENSG00000233454 | AC069285.2 |  |  | 37939 | NaN | 0.3540 | No |
| 163 | ENSG00000230833 | RPEP3 |  |  | 38288 | NaN | 0.3482 | No |
| 164 | ENSG00000277358 | AL138693.1 |  |  | 38513 | NaN | 0.3444 | No |
| 165 | ENSG00000253810 | PSAT1P1 |  |  | 38548 | NaN | 0.3439 | No |
| 166 | ENSG00000240244 | GAPDHP33 |  |  | 38751 | NaN | 0.3405 | No |
| 167 | ENSG00000225455 | TPI1P4 |  |  | 38844 | NaN | 0.3390 | No |
| 168 | ENSG00000267597 | RPIAP1 |  |  | 38925 | NaN | 0.3377 | No |
| 169 | ENSG00000249210 | GAPDHP38 |  |  | 39132 | NaN | 0.3342 | No |
| 170 | ENSG00000136872 | ALDOB |  |  | 39227 | NaN | 0.3326 | No |
| 171 | ENSG00000214041 | PGAM1P4 |  |  | 39264 | NaN | 0.3321 | No |
| 172 | ENSG00000224751 | SHMT1P1 |  |  | 39302 | NaN | 0.3315 | No |
| 173 | ENSG00000244457 | ENO1P1 |  |  | 39338 | NaN | 0.3310 | No |
| 174 | ENSG00000278419 | AL451164.3 |  |  | 39555 | NaN | 0.3273 | No |
| 175 | ENSG00000236554 | ASNSP3 |  |  | 39671 | NaN | 0.3254 | No |
| 176 | ENSG00000225017 | AP004289.1 |  |  | 39741 | NaN | 0.3243 | No |
| 177 | ENSG00000274231 | AC134878.1 |  |  | 39901 | NaN | 0.3216 | No |
| 178 | ENSG00000226196 | GAPDHP75 |  |  | 40398 | NaN | 0.3133 | No |
| 179 | ENSG00000226540 | GAPDHP73 |  |  | 40746 | NaN | 0.3074 | No |
| 180 | ENSG00000114786 | ABHD14A-ACY1 |  |  | 40751 | NaN | 0.3074 | No |
| 181 | ENSG00000214745 | AL391419.1 |  |  | 41116 | NaN | 0.3013 | No |
| 182 | ENSG00000230478 | Z96811.1 |  |  | 41260 | NaN | 0.2989 | No |
| 183 | ENSG00000277549 | AL359920.1 |  |  | 41317 | NaN | 0.2980 | No |
| 184 | ENSG00000213594 | GAPDHP25 |  |  | 41432 | NaN | 0.2961 | No |
| 185 | ENSG00000250982 | GAPDHP35 |  |  | 41512 | NaN | 0.2948 | No |
| 186 | ENSG00000225308 | ASS1P11 |  |  | 41523 | NaN | 0.2946 | No |
| 187 | ENSG00000213487 | ASS1P4 |  |  | 41566 | NaN | 0.2940 | No |
| 188 | ENSG00000243033 | GAPDHP47 |  |  | 41629 | NaN | 0.2930 | No |
| 189 | ENSG00000239775 | AC017116.1 |  |  | 41764 | NaN | 0.2907 | No |
| 190 | ENSG00000228872 | AC096664.1 |  |  | 42602 | NaN | 0.2766 | No |
| 191 | ENSG00000264236 | AC096708.1 |  |  | 42636 | NaN | 0.2761 | No |
| 192 | ENSG00000232780 | GAPDHP74 |  |  | 42735 | NaN | 0.2744 | No |
| 193 | ENSG00000236167 | GAPDHP57 |  |  | 42770 | NaN | 0.2739 | No |
| 194 | ENSG00000269968 | AC006064.4 |  |  | 42888 | NaN | 0.2720 | No |
| 195 | ENSG00000224333 | GAPDHP20 |  |  | 43162 | NaN | 0.2674 | No |
| 196 | ENSG00000236973 | GAPDHP51 |  |  | 43568 | NaN | 0.2605 | No |
| 197 | ENSG00000225477 | AC099730.1 |  |  | 43607 | NaN | 0.2599 | No |
| 198 | ENSG00000277172 | GAPDHP41 |  |  | 43750 | NaN | 0.2576 | No |
| 199 | ENSG00000251470 | ASNSP4 |  |  | 44064 | NaN | 0.2523 | No |
| 200 | ENSG00000215043 | GLULP6 |  |  | 44418 | NaN | 0.2464 | No |
| 201 | ENSG00000241861 | GAPDHP50 |  |  | 44899 | NaN | 0.2382 | No |
| 202 | ENSG00000237173 | AC099796.2 |  |  | 45550 | NaN | 0.2272 | No |
| 203 | ENSG00000229541 | GAPDHP26 |  |  | 45665 | NaN | 0.2254 | No |
| 204 | ENSG00000224962 | PSAT1P4 |  |  | 46382 | NaN | 0.2132 | No |
| 205 | ENSG00000242849 | ALDOAP1 |  |  | 46610 | NaN | 0.2094 | No |
| 206 | ENSG00000258012 | AC026111.1 |  |  | 46729 | NaN | 0.2075 | No |
| 207 | ENSG00000224678 | GAPDHP46 |  |  | 47156 | NaN | 0.2003 | No |
| 208 | ENSG00000235595 | GAPDHP23 |  |  | 47292 | NaN | 0.1980 | No |
| 209 | ENSG00000234732 | RPEP5 |  |  | 47459 | NaN | 0.1953 | No |
| 210 | ENSG00000215000 | AL034403.1 |  |  | 47719 | NaN | 0.1909 | No |
| 211 | ENSG00000227881 | ASS1P5 |  |  | 47861 | NaN | 0.1886 | No |
| 212 | ENSG00000225639 | PGAM1P2 |  |  | 47863 | NaN | 0.1886 | No |
| 213 | ENSG00000217791 | ASS1P9 |  |  | 48094 | NaN | 0.1847 | No |
| 214 | ENSG00000235868 | GAPDHP31 |  |  | 48131 | NaN | 0.1842 | No |
| 215 | ENSG00000233010 | RPEP4 |  |  | 48182 | NaN | 0.1834 | No |
| 216 | ENSG00000178596 | GAPDHP29 |  |  | 48809 | NaN | 0.1728 | No |
| 217 | ENSG00000232392 | AC002366.1 |  |  | 48848 | NaN | 0.1722 | No |
| 218 | ENSG00000224055 | GAPDHP55 |  |  | 49237 | NaN | 0.1656 | No |
| 219 | ENSG00000007350 | TKTL1 |  |  | 49335 | NaN | 0.1640 | No |
| 220 | ENSG00000248739 | AC069306.1 |  |  | 50223 | NaN | 0.1490 | No |
| 221 | ENSG00000239873 | GAPDHP27 |  |  | 50434 | NaN | 0.1455 | No |
| 222 | ENSG00000236893 | ASS1P7 |  |  | 50630 | NaN | 0.1422 | No |
| 223 | ENSG00000231739 | GAPDHP59 |  |  | 50933 | NaN | 0.1371 | No |
| 224 | ENSG00000249930 | AC007016.1 |  |  | 50944 | NaN | 0.1370 | No |
| 225 | ENSG00000232144 | PSAT1P2 |  |  | 51214 | NaN | 0.1325 | No |
| 226 | ENSG00000249018 | GAPDHP56 |  |  | 51348 | NaN | 0.1303 | No |
| 227 | ENSG00000143627 | PKLR |  |  | 51386 | NaN | 0.1297 | No |
| 228 | ENSG00000241526 | AC112771.1 |  |  | 52010 | NaN | 0.1191 | No |
| 229 | ENSG00000216352 | AL590824.1 |  |  | 52187 | NaN | 0.1162 | No |
| 230 | ENSG00000232718 | GAPDHP48 |  |  | 52438 | NaN | 0.1120 | No |
| 231 | ENSG00000225502 | GAPDHP16 |  |  | 52618 | NaN | 0.1090 | No |
| 232 | ENSG00000235306 | GAPDHP34 |  |  | 53407 | NaN | 0.0957 | No |
| 233 | ENSG00000220515 | PGAM1P10 |  |  | 53779 | NaN | 0.0894 | No |
| 234 | ENSG00000220517 | ASS1P1 |  |  | 53781 | NaN | 0.0894 | No |
| 235 | ENSG00000183663 | AC007992.1 |  |  | 54027 | NaN | 0.0853 | No |
| 236 | ENSG00000258739 | ENO1P2 |  |  | 54240 | NaN | 0.0818 | No |
| 237 | ENSG00000231907 | GAPDHP37 |  |  | 54813 | NaN | 0.0721 | No |
| 238 | ENSG00000241218 | AC063944.2 |  |  | 54966 | NaN | 0.0696 | No |
| 239 | ENSG00000204894 | AC104692.1 |  |  | 55080 | NaN | 0.0677 | No |
| 240 | ENSG00000230787 | PSAT1P3 |  |  | 55130 | NaN | 0.0669 | No |
| 241 | ENSG00000230849 | GOT2P2 |  |  | 55251 | NaN | 0.0649 | No |
| 242 | ENSG00000248488 | PGAM4P2 |  |  | 55514 | NaN | 0.0605 | No |
| 243 | ENSG00000214297 | ALDOAP2 |  |  | 55551 | NaN | 0.0599 | No |
| 244 | ENSG00000215197 | PGAM4P1 |  |  | 55939 | NaN | 0.0534 | No |
| 245 | ENSG00000254765 | AC011092.1 |  |  | 56477 | NaN | 0.0443 | No |
| 246 | ENSG00000228367 | AC108120.1 |  |  | 56934 | NaN | 0.0366 | No |
| 247 | ENSG00000250825 | PGAM1P12 |  |  | 56984 | NaN | 0.0358 | No |
| 248 | ENSG00000237256 | PGAM3P |  |  | 57133 | NaN | 0.0334 | No |
| 249 | ENSG00000272917 | AC010186.4 |  |  | 57190 | NaN | 0.0325 | No |
| 250 | ENSG00000233876 | GAPDHP68 |  |  | 57807 | NaN | 0.0221 | No |
| 251 | ENSG00000241790 | ENO1P4 |  |  | 57924 | NaN | 0.0201 | No |
| 252 | ENSG00000215057 | PKMP5 |  |  | 58035 | NaN | 0.0183 | No |
| 253 | ENSG00000186743 | TPI1P3 |  |  | 58381 | NaN | 0.0125 | No |
| 254 | ENSG00000256450 | AC007552.1 |  |  | 58514 | NaN | 0.0103 | No |
| 255 | ENSG00000250933 | GAPDHP66 |  |  | 58563 | NaN | 0.0095 | No |
| 256 | ENSG00000229937 | PRPS1L1 |  |  | 58896 | NaN | 0.0039 | No |
| 257 | ENSG00000250732 | RPEP1 |  |  | 58968 | NaN | 0.0028 | No |
| 258 | ENSG00000219930 | GAPDHP67 |  |  | 59058 | NaN | 0.0013 | No |
Table: GSEA details [plain text format]

  

Fig 2: BIOSYNTHESIS\_OF\_AMINO\_ACIDS(HSA01230)      
 Blue-Pink O' Gram in the Space of the Analyzed GeneSet

  

Fig 3: BIOSYNTHESIS\_OF\_AMINO\_ACIDS(HSA01230): Random ES distribution      
 Gene set null distribution of ES for **BIOSYNTHESIS\_OF\_AMINO\_ACIDS(HSA01230)**
